# Do Plants Need to Be Sprayed? New Insights into VOC-Mediated Biostimulation by Wood Vinegar

**DOI:** 10.3390/biology15030267

**Published:** 2026-02-02

**Authors:** Riccardo Fedeli, Stefano Loppi

**Affiliations:** 1 BioAgryLab, Department of Life Sciences, University of Siena, 53100 Siena, Italy; loppi@unisi.it; 2 National Biodiversity Future Center, 90133 Palermo, Italy

**Keywords:** gaseous signalling, pyroligneous acid, stomatal regulation, secondary metabolism, wood distillate

## Abstract

Wood vinegar (**WV**), a by-product of biomass pyrolysis rich in organic acids and phenolic compounds, has gained increasing attention as a sustainable input for crop production, mainly through foliar application. However, its high content of volatile organic compounds (VOCs) suggests that **WV** may (also) interact with plants through the gaseous phase, a pathway that has so far been overlooked. This study tested the hypothesis that WV can modulate plant physiological performance, metabolic status, and nutrient accumulation not only via direct foliar contact but also through exposure to **WV**-derived VOCs. Lettuce (*Lactuca sativa* L.) was used as a model crop and grown under controlled environmental conditions. Plants were subjected to weekly treatments consisting of either foliar spraying with a 0.2% (*v*/*v*) **WV** solution or exposure to VOCs released from the same solution in a sealed chamber, without direct contact between the liquid and plant tissues, and were compared with untreated controls. Notably, plants exposed exclusively to **WV**-derived VOCs showed responses similar to those observed following foliar application. Both treatments significantly increased fresh weight, the content of chlorophyll, total polyphenols and the accumulation of key macro- and micronutrients, including Ca, K, P, S, and Zn. For both treatments, the efficiency of photosystem II remained stable, indicating the absence of photochemical stress, while stomatal conductance, transpiration rate, intercellular CO_2_ concentration, and net photosynthetic rate were markedly reduced, suggesting a regulated stomatal response. Physiological, biochemical, and mineral parameters were assessed using non-destructive optical techniques, gas exchange measurements, spectrophotometric assays, and X-ray fluorescence analysis. These findings indicate that exposure to the volatile fraction released from WV under the exposure conditions adopted in this study can elicit biostimulant-like responses comparable to those observed after foliar application.

## Simple Summary

Wood vinegar is a natural liquid obtained during the production of charcoal and is rich in organic substances that can help plants grow. It is usually sprayed directly onto leaves, but because it also releases vapors, it may affect plants through the air as well. This study explored whether wood vinegar can improve plant growth not only by touching the leaves, but also through its vapors alone. Lettuce plants were used as a model crop and were either sprayed with a very diluted wood vinegar solution, exposed only to vapors coming from the same solution, or left untreated. The results showed that plants exposed only to the vapors grew just as well as those sprayed directly. In both cases, plants were heavier, greener, richer in beneficial plant compounds, and contained higher levels of important nutrients such as calcium, potassium, phosphorus, sulfur, and zinc. The plants also showed controlled water loss and no signs of stress. These findings show that wood vinegar vapors alone can stimulate plant growth, revealing a new way this natural product works.

## 1. Introduction

In the context of sustainable agriculture and circular bioeconomy, there has been growing interest in the valorisation of biomass by-products as a functional solution for agricultural production [1,2,3]. Among these by-products, wood vinegar (**WV**), also known as wood distillate or pyroligneous acid, has garnered attention due to its chemical composition and the reported biological effects on crops [4,5]. Wood vinegar is obtained through the condensation of vapors produced during the pyrolysis of woody biomass and consists of an aqueous solution rich in organic acids, phenolic compounds, alcohols, ketones, and other low-molecular-weight organic substances [6,7]. While the use of **WV** has primarily focused on foliar applications [8,9,10], the presence of volatile organic compounds (VOCs) suggests that its interaction with plants may also occur through the gaseous phase, without any direct contact.

Several studies have documented the potential of low concentrations (around 0.2%) of **WV** when applied to leaves by spraying [8,11,12], highlighting its capacity to increase plant growth [13], nutrient availability [14], microbial activity [15], and resistance to abiotic stress [16]. These effects have been attributed to its high content of organic acids, particularly acetic acid, and to a complex mixture of phenolic compounds [17]. Organic acids can influence intracellular pH regulation [18], enhance nutrient availability within tissues [19], and support metabolic activity [20], while phenolic compounds may function as signalling molecules or elicitors activating adaptive physiological and biochemical responses in plants [21]. However, most existing research implicitly assumes that these effects require direct contact between **WV** and plant tissues, overlooking the potential contribution of VOCs released into the surrounding environment.

Plants are highly sensitive to VOCs and can detect and respond to a wide variety of these compounds [22,23,24]. Airborne compounds emitted by plants, microorganisms, or abiotic sources can function as signalling agents, influencing plant physiology, metabolism, and defense mechanisms [25,26]. Well-documented examples include green leaf volatiles and other low-molecular-weight organic compounds that induce defense responses [27,28], prime plants against stresses [29,30], or alter growth patterns even at very low concentrations [31,32]. In this framework, the VOC fraction of **WV** represents a potentially important, yet unexplored, pathway of interaction with plants.

Chemical characterization of **WV** typically reveals a high concentration of acetic acid, along with other short-chain organic acids and a variety of phenolic compounds [17]. Acetic acid is highly volatile and readily partitions into the gas phase under ambient conditions, especially when the solution is exposed over a large surface area or maintained at elevated temperatures [33]. Recent research has demonstrated that vapor of acetic acid can influence plant physiological processes; however, in the present study the specific contribution of individual WV-derived volatile compounds was not directly assessed [34,35]. Although phenolic compounds are generally less volatile, their continuous release in closed environments may still contribute to the overall chemical atmosphere surrounding plants [36,37].

The importance of atmospheric exposure to **WV** VOCs may become particularly interesting in closed or semi-closed environments, such as greenhouses and growth chambers. Unlike conditions in the open field, where dispersion rapidly dissolves airborne compounds, enclosed environments amplify the persistence and intensity of VOC exposure [38,39]. Most available research implicitly assumes that the beneficial effects of **WV** require direct foliar contact, overlooking the potential contribution of VOCs released into the surrounding atmosphere. Moreover, foliar spraying, although widely adopted, is inherently inefficient due to substantial losses caused by evaporation, droplet drift, runoff, and uneven leaf coverage, particularly under greenhouse or field conditions. These factors reduce the actual amount of applied product reaching the target tissues, limiting the efficiency and reproducibility of **WV** treatments.

In this context, the volatile fraction of **WV** represents a completely unexplored and potentially more efficient pathway of interaction with plants, capable of bypassing the physical constraints associated with spray applications. To date, no studies have experimentally disentangled the effects of **WV**-derived VOCs from those resulting from direct foliar contact, and their relative contribution to plant physiological and metabolic responses remains unknown. Addressing this gap is particularly relevant for controlled or semi-closed environments, where VOC accumulation may enhance treatment efficiency while reducing product losses and application inputs.

Based on this evidence, the present study tested the hypothesis that **WV** can modulate plant physiological performance, biochemical status, and nutrient accumulation not only through direct foliar application but also via exposure to VOCs released into the surrounding environment. Specifically, we hypothesized that atmospheric exposure to **WV**-derived VOCs is sufficient to induce changes in photosynthetic activity, stomatal regulation, antioxidant metabolism, and mineral composition of plant tissues, comparable to those observed following foliar application, thereby indicating that direct contact between the **WV** solution and plant tissues is not required for its biological activity.

## 2. Materials and Methods

### 2.1. Wood Vinegar

Wood pyrolysis generates two main residual fractions: a solid phase, known as biochar, and a liquid phase referred to as **WV**. To enhance **WV** yield relative to biochar, fast pyrolysis represents the most effective approach and is typically performed at temperatures between 350 and 500 °C. This technique involves rapid heating of lignocellulosic biomass followed by immediate cooling. Under these conditions, the liquid fraction accounts for approximately 60–75% of the total products, while the solid fraction represents about 15–25% and the gaseous fraction 10–20%.

**WV** is mainly composed of water (80–90%) and contains more than 300 water-soluble organic compounds, including organic acids, alkanes, phenolic compounds, alcohols, and esters. It should be noted that the chemical composition of **WV** is highly variable, as it depends on both the type of feedstock and the operational parameters adopted during the pyrolysis process, resulting in products with distinct chemical properties.

The selection of the 0.2% (*v*/*v*) **WV** treatment concentration was based on evidence from previous studies and on preliminary dose–response experiments. Several works have reported that low **WV** concentrations, typically ranging between 0.1% and 0.5%, are effective in eliciting biological activity while avoiding phytotoxic effects, which may occur at higher doses due to the acidity and the presence of phenolic compounds. In particular, concentrations around 0.2% have been shown to provide a favorable balance between efficacy and safety, making them suitable for experimental applications. Therefore, the 0.2% **WV** concentration adopted in this study was selected as a representative and literature-supported dose to ensure comparability with previous findings and to minimize potential adverse effects [40,41].

The **WV** used in the present study (Distillato di Legno, produced by BioDea^®^, Arezzo, Italy [42]) was obtained from the pyrolysis of sweet chestnut [(*Castanea sativa* Mill., *Robinia pseudoacacia* L., *Fraxinus ornus* L., *Alnus glutinosa* (L.) Gaertn., and *Quercus robur* L.).] wood derived from forest management residues. This product has already been widely investigated for soil-based crop cultivation, showing significant improvements in both crop yield and nutritional quality. The chemical features of this **WV** are reported in Table S2.

### 2.2. Experimental Setup

**Figure 1:**
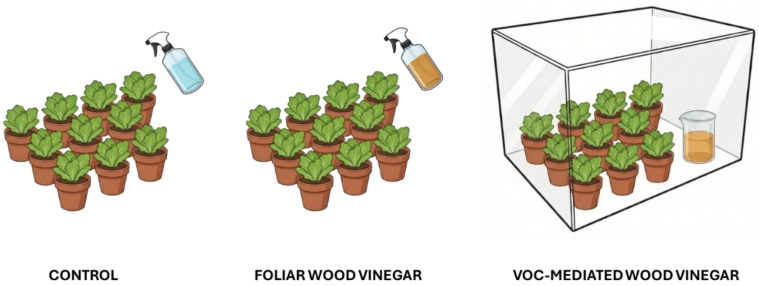
Schematic representation of the treatments.

Lettuce (*Lactuca sativa* L.) was used as a model crop plant, for its well-known reactions to foliar application of **WV** [43,44,45]. Seedlings of *L sativa* (*n* = 30), purchased from a local nursery, were initially grown in polystyrene trays under greenhouse conditions for two weeks. Plants were then transferred to the laboratory and transplanted into plastic pots (10 × 10 × 10 cm) filled with commercial potting soil (VigorPlant Srl, Piacenza, Italy; characteristics are reported in Fedeli et al. [16] and in Table S1). Following transplantation, seedlings were allowed to acclimate for one week in a controlled-environment growth chamber set at 20 ± 1° C, 70 ± 2% relative humidity, a photosynthetic photon flux density of 400 μmol m^−2^ s^−1^, and a 16/8 h photoperiod. Subsequently, plants were subjected to three different treatments (10 plants per treatment) applied once per week: (*i*) control plants sprayed with deionized water (**C**); (*ii*) plants treated with a 0.2% **WV** solution applied by foliar spraying (**F-WV**); and (*iii*) plants sprayed with deionized water and exposed to VOCs released from a 0.2% **WV** solution placed inside a closed chamber (**VOC-WV**) (Figure 1).

The chamber-based exposure system was adopted to specifically evaluate the effects of **WV**-derived VOCs while excluding any direct contact between the liquid solution and plant tissues. The exposure chamber consisted of a rigid, transparent enclosure (40 × 20 × 20 cm; total internal volume ≈ 16 L), constructed to allow full sealing during the exposure period. This approach allowed the volatile fraction of **WV** to be assessed independently from foliar uptake under controlled conditions. For VOC generation, 250 mL of a 0.2% **WV** solution were placed inside the chamber in an open 250 mL glass beaker, allowing passive volatilization. The exposed liquid surface corresponded to the open beaker mouth. The chamber was kept fully sealed throughout the 5 h exposure period, with no active air exchange, in order to allow VOC accumulation in the headspace.

The exposure duration was set at 5 h to ensure sufficient accumulation of **WV**-derived VOCs in the chamber atmosphere while avoiding prolonged confinement that could induce secondary effects unrelated to VOC exposure, such as alterations in humidity, gas composition, or plant microclimate. The exposure window (9:00–13:00) was selected to coincide with the central part of the photoperiod, when stomatal conductance and leaf gas exchange are typically high under controlled environmental conditions. This timing is commonly adopted in plant physiological studies to minimize diurnal variability and to investigate gas-mediated processes under stable and physiologically active conditions [46,47,48].

During the exposure period, temperature, relative humidity, and light conditions inside the chamber were the same as those set for the controlled-environment growth chamber (20 ± 1 °C, 70 ± 2% RH, PPFD 400 μmol m^−2^ s^−1^), as the sealed chamber was placed directly within the growth chamber.

Specifically, for the **VOC-WV** treatment, plants were placed inside a sealed exposure chamber, allowing passive release of VOCs. Exposure was carried out for 5 h, from 9:00 to 13:00, without direct contact between the solution and the plant tissue. After the treatments, all 30 pots were positioned again inside the controlled-environment growth chamber and randomly rotated every two days to reduce potential micro-environmental variability. This rotation procedure was adopted to minimize spatial heterogeneity in light distribution, air circulation, temperature, and humidity within the growth chamber, thereby limiting microclimate-related effects on plant growth and physiology. At the end of the experiment (5 weeks after transplantation), the fresh weight of the above-ground part of the plants was recorded using a professional balance.

### 2.3. Photosynthetic Parameters

The photosynthetic parameters were recorded before the harvest of the plants. Specifically, the total chlorophyll content was determined using a non-destructive chlorophyll content meter (CCM-300, Opti-Science, Hudson, IN, USA [49]). Measurements were performed on the apical regions of the three fully expanded leaves of each plant, avoiding the main nerves [50]. Chlorophyll content values were expressed on a leaf surface basis (mg/cm^2^). For chlorophyll fluorescence analysis, plants were dark-adapted for 15 min and subsequently exposed to a 1-s saturating pulse of red light (650 nm) at an intensity of 2400 μmol m^−2^ s^−1^ [51]. Chlorophyll fluorescence was recorded using a plant efficiency analyzer (Handy PEA, Hansatech Ltd., Norfolk, UK [52]). The maximum quantum efficiency of PSII (Fv/Fm) and the performance index (PI_abs_) were used as indicators of photosynthetic performance and plant physiological status.

### 2.4. Gas Exchange Parameters

Leaf gas exchange measurements were carried out before plant harvest on fully developed leaves using a LI-6800 portable photosynthesis system (LI-COR Inc., Lincoln, NE, USA). A single measurement was taken from each plant, on a randomly selected fully expanded leaf. Measurements were performed between 11:00 and 14:00. During measurements, CO_2_ concentration inside the instrument chamber was maintained at 400 μmol mol^−1^ and air flow was set to 600 μmol s^−1^ [53]. Gas exchange parameters were recorded once steady-state conditions were achieved, as indicated by stable CO_2_ and H_2_O fluxes. The parameters measured included net CO_2_ assimilation rate (A), intercellular CO_2_ concentration (Ci), stomatal conductance (gsw), and transpiration rate (E).

### 2.5. Antioxidant Compounds

Plant samples were cut into small pieces, oven-dried at 40 ° C for 24 h, and finely ground using an Ultra-Turrax homogenizer (IKA A10, IKA-Werke GmbH & Co. KG, Staufen im Breisgau, Germany). Approximately 0.5 g of dried material were extracted with 5 mL of 80% methanol (1:10 ratio) by homogenization for 30 min, and the extracts were subsequently stored at 4 °C in the dark for 48 h [54]. After extraction, samples were filtered through Whatman No. 1 filter paper to obtain clear extracts for antioxidants quantification.

The total polyphenol content was determined using a modified Folin–Ciocalteu colorimetric assay, following the method reported by Fedeli et al. [55]. Briefly, an aliquot of extract was mixed with distilled water and Folin–Ciocalteu reagent, followed by the addition of sodium carbonate, and incubated in the dark for 90 min before measuring absorbance at 760 nm with a UV–Vis spectrophotometer (Agilent 8453, Santa Clara, CA, USA). The concentration of total polyphenols was calculated using a gallic acid calibration curve (5–300 μg mL^−1^) and expressed as milligrams of gallic acid equivalents per gram of dry weight (mg GAE g^−1^ DW).

The total flavonoid content was assessed using an aluminum chloride-based colorimetric method, following the method reported by Carullo et al. [56]. An aliquot of the above extract was sequentially reacted with sodium nitrite, aluminum chloride, and sodium hydroxide, with incubation steps carried out in the dark. Absorbance was recorded at 415 nm using a UV-VIS spectrophotometer (Agilent 8453, Santa Clara, CA, USA), and the concentration of total flavonoids was quantified against a quercetin standard curve (12.5–150 μg mL^−1^), with results expressed as milligrams of quercetin equivalents per gram of dry weight (mg QE g^−1^ DW).

### 2.6. Mineral Content

The mineral content was determined with a portable X-ray fluorescence (XRF) analyzer (Olympus Vanta Series C, Olympus Corp., Waltham, MA, USA; [57]). The instrument was equipped with a silver-anode X-ray tube operating within an excitation energy range of 15–40 kV and a large-area silicon drift detector, following previously described procedures [58]. Approximately 1 g of finely ground oven-dried plant material was loaded into plastic sample cups and for each sample, three consecutive measurements were performed, with an acquisition time of 20 s per beam. The elements quantified included calcium (Ca), copper (Cu), iron (Fe), potassium (K), manganese (Mn), phosphorus (P), sulfur (S), and zinc (Zn). Method accuracy was verified using 14 certified plant reference materials [59]. All results were reported on a dry weight basis.

### 2.7. Statistical Analysis

Data distribution was first evaluated using the Shapiro–Wilk test to assess normality. As normality assumptions were not met (*p* > 0.05), differences among treatments were analyzed using the non-parametric Kruskal–Wallis ANOVA, followed by Dunn’s post hoc test for multiple comparisons. Results are presented as median values ± error, where the error represents a robust dispersion estimate calculated as the median absolute deviation (MAD) divided by the square root of the number of observations, and is intended as an indicator of variability around the median rather than as a parametric standard error. Statistical significance was accepted at *p* < 0.05. Statistical analysis was performed using R software (Version 2026.01.0+392) [60].

## 3. Results and Discussion

### 3.1. Fresh Weight and Photosynthetic Parameters

**Figure 2:**
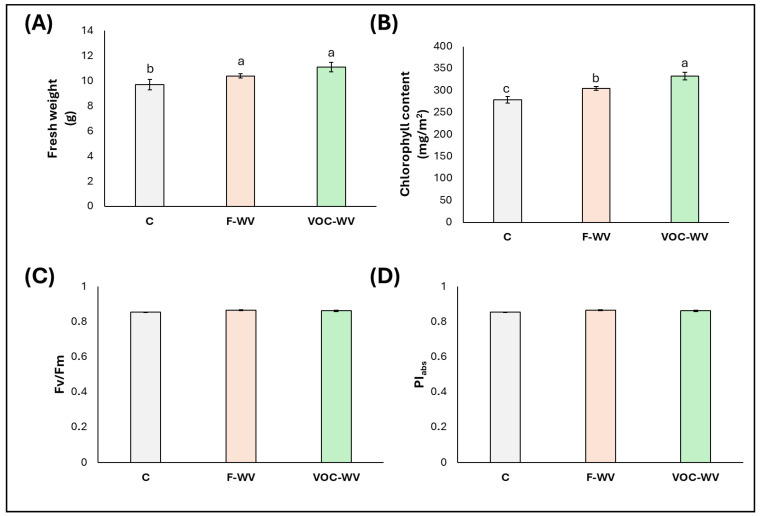
Biometric and photosynthetic parameters (median ± error). (**A**). fresh weight, (**B**). chlorophyll content, (**C**). Fv/Fm, (**D**). PI_abs_. **C** = plants untreated with WV; **F-WV** = plants foliar-sprayed with 0.2% wood vinegar; **VOC-WV** = plants exposed to VOCs from 0.2% WV. Different letters indicate statistically significant (*p* < 0.05) differences between treatments.

Fresh weight and chlorophyll content were significantly influenced by the treatments, showing a significant increase in both **F-WV** (+13% and +8%, respectively) and **VOC-WV** plants (+16% and +18%, respectively) compared with **C** (Figure 2A,B). It is noteworthy that the chlorophyll content of **VOC-WV** plants was even higher than that of **F-WV** plants. An increase in chlorophyll content does not necessarily translate into higher net photosynthetic rates, particularly when stomatal conductance is concurrently reduced. In this context, enhanced chlorophyll accumulation may reflect an acclimatory or compensatory adjustment aimed at maintaining light-harvesting capacity under conditions of restricted CO_2_ diffusion, rather than an immediate enhancement of carbon assimilation. Similar dissociations between chlorophyll content and photosynthetic performance have been reported in plants exhibiting regulated stomatal limitation or acclimation responses. In contrast, no significant differences were detected among treatments for the maximum quantum efficiency of PSII (Fv/Fm) or the performance index (PI_abs_), which remained stable across all treatments (Figure 2C,D).

The increase in fresh weight and chlorophyll content, together with the absence of changes in PSII photochemical efficiency, indicates that **WV** promoted plant growth and leaf pigment accumulation without inducing photosynthetic stress. Stable values of Fv/Fm and PI_abs_ across treatments suggest that the photosynthetic apparatus remained fully functional, with PSII operating within the optimal range typical of healthy, non-stressed plants [61,62,63]. This response pattern is consistent with previous studies on foliar applications of **WV** at low concentrations [51,64,65], which have reported enhanced biomass accumulation and chlorophyll content without detrimental effects on chlorophyll fluorescence parameters, supporting the interpretation of **WV** as a mild biostimulant rather than a stress-inducing agent [53,66].

In the absence of direct contact, the comparable response observed in **VOC-WV** plants suggests that this treatment may elicit growth-related responses independently of foliar treatment. Although no studies have explicitly investigated the effects of **WV**-derived VOCs, similar results have been reported for VOCs from different sources, including volatiles emitted by plants and microbes, which are known to enhance leaf development and chlorophyll accumulation without affecting PSII efficiency [67,68,69]. Volatile organic acids and other low-molecular-weight airborne compounds have been shown to act as signaling molecules in plants; however, direct identification of the compounds involved in the present system was not performed [70,71].

### 3.2. Gas Exchange Parameters

**Figure 3:**
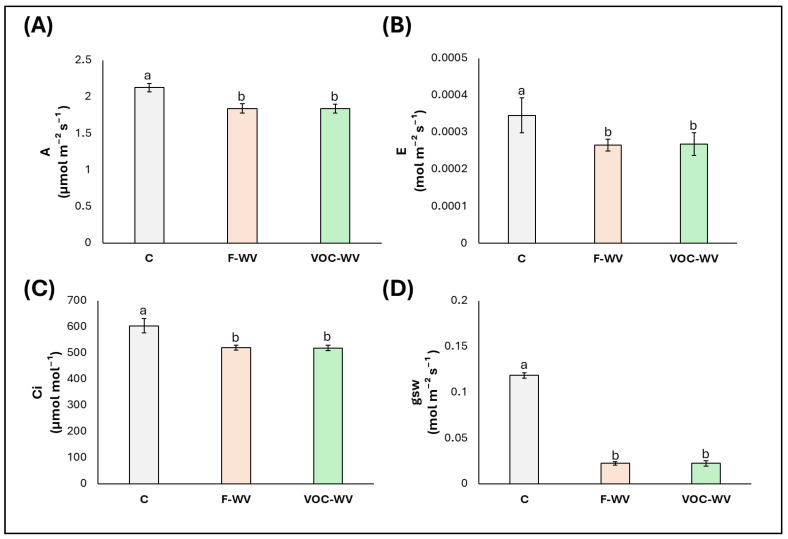
Leaf gas exchange parameters (median ± error). (**A**). net CO_2_ assimilation rate (A), (**B**). transpiration rate (E), (**C**). intercellular CO_2_ concentration (Ci), (**D**). stomatal conductance (gsw). **C** = plants untreated with WV; **F-WV** = plants foliar-sprayed with 0.2% wood vinegar; **VOC-WV** = plants exposed to VOCs from 0.2% wood vinegar. Different letters indicate statistically significant (*p* < 0.05) differences between treatments.

All gas exchange parameters were significantly reduced by the treatments (Figure 3) compared to the control, with reductions in the range −14% and −17%, in F-WV and VOC-WV plants, respectively, for net photosynthetic rate (Figure 3A), −32% and −35%, for transpiration rate (Figure 3B), −15% and −12% for intercellular CO_2_ concentration (Figure 3C), and −80% and −77% for stomatal conductance (Figure 3D).

The strong and consistent reduction in stomatal conductance and transpiration rate observed in both **F-WV** and **VOC-WV** plants indicates that **WV** treatments primarily affect stomatal regulation rather than the photochemical capacity of the photosynthetic apparatus. The concurrent decrease in Ci is compatible with a contribution of stomatal limitation to the observed reduction in A; however, Ci alone does not allow a definitive discrimination between stomatal and non-stomatal limitations. Importantly, the absence of changes in Fv/Fm and PI_abs_ indicates that photochemical efficiency was maintained, supporting the interpretation of a regulated physiological adjustment rather than a stress-induced impairment of the photosynthetic apparatus [72]. Although the magnitude of stomatal conductance reduction was substantial, the maintenance of PSII efficiency and the absence of photochemical impairment suggest that this response reflects a strong but regulated adjustment of gas exchange, rather than a collapse of photosynthetic function typically associated with severe stress.

Similar stomatal responses have been reported in previous studies investigating foliar applications of **WV** or acetic acid–containing solutions, where reductions in stomatal conductance and transpiration were associated with improved water-use regulation and enhanced tolerance to abiotic stress conditions [53,73]. In these contexts, organic acids have been proposed to influence guard cell behavior either directly, through pH-related effects, or indirectly, via signalling pathways involved in stomatal control [71,74].

Although no studies have directly examined the effects of **WV**-derived VOCs on plant gas exchange, comparable responses have been widely documented for VOCs of different origins. Volatile organic acids and other low-molecular-weight airborne compounds have been shown to induce partial stomatal closure, reduce transpiration, and modulate photosynthetic rates without causing photoinhibition, often as part of a priming or acclimation response [73,75,76]. Such effects are frequently interpreted as adaptive adjustments that balance carbon gain with water conservation under changing environmental conditions [77,78].

Although the present study was not designed to directly investigate molecular, hormonal, or redox-related signaling pathways, the coordinated changes observed in stomatal conductance, transpiration, and CO_2_ assimilation indicate a regulated physiological adjustment rather than a collapse of photosynthetic function. However, it must be emphasized that no direct measurements of phytohormones, redox status, or gene expression were performed, and therefore any mechanistic interpretation remains purely inferential.

Previous studies have shown that exposure to volatile organic acids, such as acetic acid, can influence stomatal behavior and stress-related physiological responses in plants [34,35]. In this context, the physiological patterns observed here are consistent with regulatory responses reported in the literature, but the present dataset does not allow attribution of the observed effects to specific signaling molecules or pathways.

Consequently, references to potential hormonal or redox-mediated mechanisms should be interpreted as conceptual frameworks derived from existing studies rather than as direct evidence from the present work. Future investigations integrating targeted analyses of hormonal profiles, redox markers, and molecular responses will be required to elucidate the mechanistic basis underlying the physiological effects observed here.

In particular, the partial stomatal closure observed in both **F-WV** and **VOC-WV**, in the absence of photochemical impairment, is consistent with signaling pathways involving abscisic acid or other regulators of guard cell function, which mediate stomatal responses under non-stressful or primed conditions. Phenolic compounds, although less volatile, may also contribute to signaling processes in closed environments, where prolonged exposure could allow biologically relevant concentrations to accumulate. Such compounds have been reported to interact with redox signaling and hormone-mediated regulatory networks, thereby influencing stomatal regulation and secondary metabolism.

Taken together, these considerations support the hypothesis that **WV**-derived VOCs may act as airborne signaling cues, triggering hormone-related regulatory pathways that modulate stomatal behavior and metabolic allocation without inducing stress-related damage. While the identification of specific signaling molecules and downstream gene expression responses requires targeted molecular analyses, the physiological patterns observed in this study provide a framework for future investigations aimed at dissecting the hormonal and genetic basis of **WV**-mediated biostimulant effects.

### 3.3. Antioxidant Compounds

**Figure 4:**
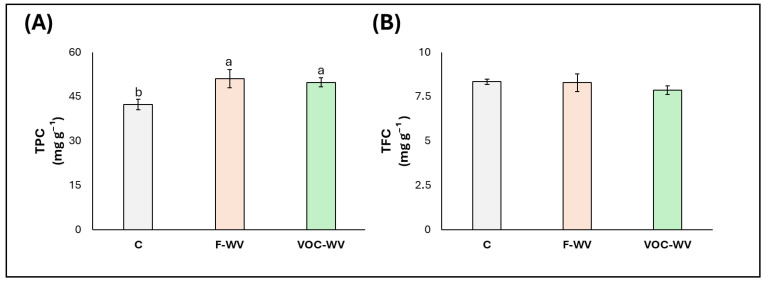
Antioxidant compounds (median ± error). (**A**). Total polyphenol content (TPC), (**B**). Total flavonoid content (TFC). **C** = plants untreated with WV; **F-WV** = plants foliar-sprayed with 0.2% wood vinegar **VOC-WV** = plants exposed to VOCs from 0.2% wood vinegar. Different letters indicate statistically significant (*p* < 0.05) differences between treatments.

The total polyphenol content was significantly increased by the treatments, with significant increases in both **F-WV** (+25%) and **VOC-WV** plants (+30%) compared with **C** (Figure 4A). Differently, TFC was not significantly influenced by the treatments (Figure 4B).

The increase in TPC, in the absence of significant changes in TFC, suggests that **WV** treatments activated specific secondary metabolism pathways rather than inducing a generalised production of phenolic compounds. This pattern is consistent with previous studies on foliar applications of **WV**, which have reported increases in TPC associated with enhanced antioxidant capacity and stress-related metabolic adjustments, often without parallel increases in all phenolic subclasses [12,79,80]. Such responses have been interpreted as indicative of a mild elicitation (eustress) effect, where plants upregulate protective compounds without entering a fully stressed state [66].

The comparable response observed in **VOC-WV** plants further supports the hypothesis that VOCs released from **WV** can act as metabolic signals capable of inducing antioxidant-related pathways. Although direct evidence for **WV**-derived VOCs is currently lacking, similar effects have been widely documented for VOCs of different origin, including plant- and microbe-emitted volatiles, which are known to trigger phenylpropanoid metabolism and polyphenol accumulation through signalling or priming mechanisms [27,69,81]. In addition, volatile organic acids and other low-molecular-weight compounds have been shown to modulate redox homeostasis and activate antioxidant defenses even at low atmospheric concentrations [82].

### 3.4. Mineral Content

The concentration of several mineral elements resulted significantly increased by the treatments compared to the control (Table 1), with increases in the range +30% and +31% in **F-WV** and **VOC-WV** plants, respectively, for Ca, +33% and +40% for K, +23% and +50% for P, +38% and +33% for S. Zinc was higher only in **VOC-WV** plants (+18%), compared to the control, while **F-WV** plants showed intermediate values and were not significantly different from either group, while Al, Cu, Fe, and Mn were not significantly influenced by the treatments.

The results of the mineral content indicate that **WV** treatments influenced nutrient accumulation in a regulated and element-specific manner, rather than causing a generalized increase in mineral content. The enhancement of Ca, K, P, and S in both treatments suggests that **WV** promotes the accumulation of key macronutrients involved in structural integrity, osmotic regulation, energy metabolism, and redox homeostasis [83,84]. Similar increases in macronutrient content have been reported in studies employing foliar applications of **WV**, where such effects have been attributed to improved nutrient use efficiency and metabolic demand associated with enhanced growth and physiological activity [79,85].

The results observed in **VOC-WV** plants further support the hypothesis that VOCs from **WV** can modulate nutrient dynamics, even in the absence of direct foliar application. This response is consistent with the changes in stomatal regulation, transpiration, and metabolic activity observed in the present study, which can influence nutrient allocation and accumulation within plant tissues [83,86]. Particularly, the increase in K and Ca may reflect adjustments in ionic balance and signalling processes [86,87], while elevated P and S levels are compatible with increased requirements for energy-related compounds and S-containing metabolites involved in stress protection, supporting the hypothesis of the eustress theory [88,89].

### 3.5. Chamber vs. Foliar Application: Implications for Wood Vinegar Mode of Action

A key aspect of the present study is the direct comparison between the effects of the same **WV** concentration (0.2%) applied through conventional foliar spraying and those induced by exposure to **WV**-derived VOCs in a closed chamber. This comparison is particularly relevant because it allows the biological activity of **WV** to be freed from the physical constraints associated with spray-based applications. Although foliar spraying is the most commonly adopted method for WV application [16,53,66], its efficiency is strongly influenced by losses due to evaporation, droplet drift, runoff, and uneven deposition on leaf surfaces, which may substantially reduce the fraction of applied product that effectively interacts with plant tissues.

The chamber treatment represents a conceptual shift, as it isolates the volatile fraction of **WV** and enables a more controlled and reproducible exposure of plants to bioactive compounds, independent of spray efficiency. The observation that plants exposed exclusively to **WV**-derived VOCs exhibited responses comparable to, or in some cases even stronger than, those observed following foliar application highlights that direct liquid contact is not a prerequisite for **WV** biological activity. This finding is particularly significant because it suggests that a substantial portion of the biostimulant effect attributed to foliar **WV** applications may be mediated by its volatile components rather than by leaf surface interactions alone.

The urgency of comparing chamber and non-chamber treatments lies in the increasing relevance of controlled and semi-closed cultivation systems, such as greenhouses, vertical farms, and growth chambers, where airborne compounds can persist and accumulate more effectively than in open-field conditions. In such environments, VOC-mediated application strategies could reduce product losses, improve application uniformity, and lower the amount of input required to achieve a physiological response, thereby enhancing both economic and environmental sustainability. By demonstrating that **WV**-derived VOCs alone are sufficient to trigger growth- and metabolism-related responses, this study provides a mechanistic basis for rethinking **WV** application strategies beyond conventional spraying.

## 4. Conclusions

This study demonstrates that the biostimulant activity of wood vinegar is not restricted to direct foliar application but can also be mediated by its volatile organic compounds. By experimentally separating foliar and gaseous exposure pathways, we show that atmospheric exposure to the volatile fraction released from WV under enclosed conditions was sufficient to reproduce most of the physiological and biochemical responses observed following foliar spraying.

Both treatments increased biomass and chlorophyll content while maintaining photosystem II efficiency, indicating the absence of photochemical stress. The concomitant reduction in stomatal conductance, transpiration, and CO_2_ assimilation suggests a regulated physiological adjustment rather than stress-induced impairment, consistent with a mild elicitor effect.

Overall, these findings identify the volatile fraction of **WV** as an active and previously overlooked component of its mode of action, thereby refining the current understanding of **WV**–plant interactions and providing a mechanistic framework for interpreting its biostimulant effects.

### Future Research Directions

Future research should aim to further elucidate the molecular and biochemical mechanisms underlying the observed physiological responses to **WV**, with particular attention to the role of specific VOCs and their interaction with hormone-mediated signaling pathways. The characterization of the gaseous phase of **WV** using analytical techniques, together with targeted analyses of antioxidant enzymes and stress-related genes, would help clarify whether VOC exposure induces a priming effect that enhances plant resilience to abiotic stresses. It should be noted that the present study did not include direct chemical characterization or quantification of VOCs accumulated in the chamber atmosphere. Therefore, attribution of the observed responses to specific volatile compounds remains actually unknown and represents a key objective for future researches.

## Figures and Tables

**Table 1:** Mineral content (median ± error). **C** = plants untreated with WV; **F-WV** = plants foliar sprayed with 0.2% wood vinegar; **VOC-WV** = plants exposed to VOCs from 0.2% wood vinegar. Different letters indicate statistically significant (*p* < 0.05) differences between treatments.

	C	F-WV	VOC-WV
**Al**	793 ± 33	872 ± 90	935 ± 63
**Ca**	7682 ± 705 ^b^	9982 ± 944 ^a^	10,031 ± 418 ^a^
**Cu**	3.5 ± 0.4	3.6 ± 0.2	3.2 ± 0.4
**Fe**	94 ± 60	76 ± 35	123 ± 39
**K**	29,491 ± 2055 ^b^	39,205 ± 3459 ^a^	41,151 ± 3188 ^a^
**Mn**	109 ± 14	120 ± 9	109 ± 9
**P**	2643 ± 214 ^b^	3257 ± 331 ^a^	3952 ± 306 ^a^
**S**	869± 32 ^b^	1203 ± 159 ^a^	1158 ± 133 ^a^
**Zn**	22.4 ± 1.2 ^b^	25.8 ± 2.2 ^ab^	26.5 ± 1.0 ^a^

## Data Availability

Data are available on reasonable request by the corresponding author.

## Supplementary Materials

The following supporting information can be downloaded at: https://www.mdpi.com/article/10.3390/biology15030267/s1, Table S1: Physiochemical characteristics of the growing medium used; Table S2: Main chemical features of wood vinegar.
