# Supplementary material for: Do Plants Need to Be Sprayed? New Insights into VOC-Mediated Biostimulation by Wood Vinegar
Source: Biology (Basel). 2026 Feb 2;15(3):267. doi: 10.3390/biology15030267 (PMC12896855; doi:10.3390/biology15030267)
Supplement: Supplementary file 1 [file biology-15-00267-s001.zip › biology-4112767-supplementary.pdf]

Table S1. Physiochemical characteristics of the growing medium used. Abbreviations: EC: electrical conductivity, CEC: cation exchange capacity; DW: dry weight.

pH	5.30 ± 0.03
EC (mS cm^{-1})	1.12 ± 0.01
CEC (meq 100 g^{-1} DW)	56.89 ± 2.67
Porosity (%)	92
Moisture content (%)	43
Ca (mg kg^{-1} DW)	23159 ± 296
Mg (mg kg^{-1} DW)	2846 ± 22
Na (mg kg^{-1} DW)	1379 ± 19
K (mg kg^{-1} DW)	1198 ± 17
P (mg kg^{-1} DW)	614 ± 14
S (mg kg^{-1} DW)	1410 ± 141
Fe (mg kg^{-1} DW)	1097 ± 10
Mn (mg kg^{-1} DW)	31 ± 1
Cu (mg kg^{-1} DW)	23 ± 1
Zn (mg kg^{-1} DW)	38 ± 1
Mo (mg kg^{-1} DW)	0.89 ± 0.01

Table S2. Main chemical features of wood vinegar.

**Parameter**	**Value**	**Method**
TOC (% DW)	58. 03	CHNS Elemental Analysis
TN (% DW)	1. 06	CHNS Elemental Analysis
H (% DW)	7. 27	CHNS Elemental Analysis
S (% DW)	0. 07	CHNS Elemental Analysis
pH	4. 00	UNI EN ISO 10523 : 2012
Density (g mL^{-1})	1. 05	
Flash point (° C)	> 60	ASTM D6450 - 16°
Total organic compounds (g L^{-1})	33. 8	
Acidity (mg L^{-1})	1289. 0	APAT CNR IRSA 2010 B Man 29 2003
Organic acids (mg L^{-1})	32. 3	
Acetic acid (mg L^{-1})	21. 5	
Polyphenols (g L^{-1})	24. 5	
Phenols (g L^{-1})	3. 00	
PCBs (mg L^{-1})	< 0. 2	CNR IRSA 24b Q 64 Vol 3 1988
Hydrocarbons C < 12 (mg L^{-1})	< 0. 1	EPA 5021A 2014 + EPA 8015D 2003
Hydrocarbons C10 – C40 (mg L^{-1})	< 0. 1	UNI EN ISO 9377 – 2 : 2002
**16 US - EPA PAHs (mg L^{-1})**		EPA 3550C 2007 + EPA 8310 1986
Acenaphthene	< 0. 05	
Acenaphthylene	< 0. 05	
Anthracene	< 0. 05	
Benzo[a]anthracene	< 0. 05	
Benzo[a]pyrene	< 0. 05	
Benzo[b]fluoranthene	< 0. 05	
Benzo[g,h,i]perylene	< 0. 05	
Benzo[k]fluoranthene	< 0. 05	
Chrysene	< 0. 05	
Dibenz[a,h]anthracene	< 0. 05	
Fluoranthene	< 0. 05	
Fluorene	< 0. 05	

Indeno [1,2,3 -cd] pyrene	< 0. 05	
Naphthalene	< 0. 05	
Phenanthrene	< 0. 05	
Pyrene	< 0. 05	
**Macronutrients (mg L $^{-1}$)**		Alkaline melting + ICP - MS analysis
Ca	325. 50	
K	23. 49	
Mg	6. 79	
P	7. 28	
**Micronutrients (mg L $^{-1}$)**		Alkaline melting + ICP - MS analysis
Cu	0. 18	
Fe	21. 16	
Mn	0. 58	
Mo	0. 0007	
Zn	3. 22	
**Other nutrients**		Alkaline melting + ICP - MS analysis
Al	1. 96	
Ba	0. 06	
Cr	0. 03	
Na	103. 59	

TOC : total organic carbon. **TN** : total nitrogen. **PCBs** : polychlorinated biphenyls. **16 US - EPA PAHs** : list of 16 priority polycyclic aromatic hydrocarbons as classified by the United State Environmental Protection Agency. **Al**: aluminum; **Ba** : barium; **C** : carbon; **Ca** : calcium; **Cr** : chromium; **Cu** : copper; **Fe** : iron; **K** : potassium; **Mg** : magnesium; **Mn** : manganese; **Mo** : molybdenum; **N** : nitrogen; **Na** : sodium; **Zn** : zinc.
